# Oral Bisphosphonates and Risk of Subtrochanteric or Diaphyseal Femur Fractures in a Population-Based Cohort

**DOI:** 10.1002/jbmr.288

**Published:** 2010-11-18

**Authors:** Seo Young Kim, Sebastian Schneeweiss, Jeffrey N Katz, Raisa Levin, Daniel H Solomon

**Affiliations:** 1 Division of Pharmacoepidemiology and Pharmacoeconomics, Brigham and Women's HospitalBoston, MA, USA; 2 Division of Rheumatology, Allergy and Immunology, Brigham and Women's HospitalBoston, MA, USA; 3 Department of Epidemiology, Harvard School of Public HealthBoston, MA, USA; 4 Department of Orthopedic Surgery, Brigham and Women's HospitalBoston, MA, USA

**Keywords:** BISPHOSPHONATES, CALCITONIN, RALOXIFENE, FEMORAL FRACTURES, OSTEOPOROSIS, SIDE EFFECTS

## Abstract

Bisphosphonates are the primary therapy for postmenopausal and glucocorticoid-induced osteoporosis. Case series suggest a potential link between prolonged use of bisphosphonates and low-energy fracture of subtrochanteric or diaphyseal femur as a consequence of oversuppression of bone resorption. Using health care utilization data, we conducted a propensity score–matched cohort study to examine the incidence rates (IRs) and risk of subtrochanteric or diaphyseal femur fractures among oral bisphosphonate users compared with raloxifene or calcitonin users. A Cox proportional hazards model evaluated the risk of these fractures associated with duration of osteoporosis treatment. A total of 104 subtrochanteric or diaphyseal femur fractures were observed among 33,815 patients. The estimated IR of subtrochanteric or diaphyseal femur fractures per 1000 person-years was 1.46 [95% confidence interval (CI) 1.11–1.88] among the bisphosphonate users and 1.43 (95% CI 1.06–1.89) among raloxifene/calcitonin users. No significant association between bisphosphonate use and subtrochanteric or diaphyseal femur fractures was found [hazard ratio (HR) = 1.03, 95% CI 0.70–1.52] compared with raloxifene/calcitonin. Even with this large study size, we had little precision in estimating the risk of subtrochanteric or diaphyseal femur fractures in patients treated with bisphosphonates for longer than 5 years (HR = 2.02, 95% CI 0.41–10.00). The occurrence of subtrochanteric or diaphyseal femur fracture was rare. There was no evidence of an increased risk of subtrochanteric or diaphyseal femur fractures in bisphosphonate users compared with raloxifene/calcitonin users. However, this study cannot exclude the possibility that long-term bisphosphonate use may increase the risk of these fractures. © 2011 American Society for Bone and Mineral Research.

## Introduction

Bisphosphonates decrease bone turnover and increase bone mineral density (BMD) by inhibiting osteoclast-mediated bone resorption.(1) Because of their clinical efficacy in reducing the risk of fractures in patients with osteopenia or osteoporosis, bisphosphonates have been used widely for the prevention and treatment of osteoporosis.(1,2) Over the past few years, a number of case series have suggested a potential association between low-energy atypical fracture of the femur and bisphosphonates use.(3–11) It is thought that long-term treatment with bisphosphonates may result in adynamic brittle bone, leading to atypical fractures, usually defined as subtrochanteric or diaphyseal femur fractures after minimal or no trauma.(8,12) Characteristic radiographic patterns of these fractures include bilateral cortical thickening and a transverse or oblique (≤30 degrees) fracture with a beaking of the cortex.(6,13)

A case series from Sweden estimated that the crude incidence of stress fractures of the femoral shaft in bisphosphonate users was 1 per 1000 person-years, 50 times higher than that for the untreated women.(11) However, these estimates were based on a small number of patients, a short follow-up duration, and uncertain denominators.

A Danish cohort study of 15,561 patients with baseline fracture reported that the hazard ratio (HR) was 1.46 [95% confidence interval (CI) 0.91–2.35] for subtrochanteric or diaphyseal femur fracture and 1.45 (95% CI 1.21–1.74) for classic osteoporotic hip fracture in alendronate users compared with no osteoporosis treatment.(14) The authors suggested that subtrochanteric or diaphyseal femur fractures were more likely related to osteoporosis than to alendronate use.(14) The results of the study should be interpreted with caution because the patients were not allocated randomly to the two groups, alendronate or no treatment. In the observational setting, there is always a reason why some patients received a prescription and some did not.(15–17) Therefore, the outcomes of the patient groups would not be comparable, and the validity of any inferences drawn about the relative effects of treatment would be subject to unmeasured confounding (ie, confounding by indication).(15,17,18) Data from recent secondary analyses using three large placebo-controlled, randomized clinical trials (RCTs) of bisphosphonates showed that the occurrence of atypical subtrochanteric or diaphyseal femoral fracture was rare among 14,195 women (0.23 per 1000 person-years).(19) Of those, 3673 were treated with alendronate and 3875 with intravenous zoledronic acid. The HRs for bisphosphonate use compared with placebo ranged from 1.03 to 1.50 with wide 95% CIs including the null value of 1 owing to the small number of outcomes. Furthermore, the generalizability of the results from clinical trials may be limited.(19) In March 2010, the US Food and Drug Administration (FDA) issued a safety announcement that there was no clear connection between bisphosphonate use and risk of atypical femur fractures in their ongoing review.(20)

Given the limitations in the currently available data, we conducted a large population-based cohort study (1) to estimate the incidence rates (IRs) and HRs of subtrochanteric and diaphyseal femoral fractures in elderly patients treated with oral bisphosphonates compared with those treated with either raloxifene or calcitonin nasal spray and (2) to examine the potential risk of these fractures associated with treatment duration. To control confounding by indication to a large extent, we used the propensity score–matching method embedded in a new user cohort design comparing two active treatments. A *propensity score* is the estimated probability of starting treatment A versus starting treatment B based on preexisting patient characteristics.(17,21) Propensity score matching has been used increasingly as an effective way to adjust a large number of confounders simultaneously, even if the outcome is rare.(17,18)

## Methods

### Data source and study patients

A large cohort study was conducted using health care utilization databases from two US states: (1) Medicare beneficiaries enrolled in the Pharmaceutical Assistance Contract for the Elderly in Pennsylvania from January 1996 through December 2006 and (2) Medicare beneficiaries enrolled in the Pharmaceutical Assistance to the Aged and Disabled in New Jersey from January 1996 through December 2006. Both drug benefits programs provided comprehensive pharmacy coverage with a small or no copayment for the low-income elderly.

We identified subjects who had at least one prescription filled for osteoporosis treatment (ie, oral bisphosphonates, raloxifene, or calcitonin nasal spray) and at least one medical claim during each of three consecutive 6-month periods before the first use of osteoporosis treatment. These criteria ensured their continuous eligibility for at least one year prior to study entry to permit us to identify new users of osteoporosis drugs and to assess their comorbidities and other medications. Propensity score–matching methods then were used to select a subset of oral bisphosphonate users and a combined group of either raloxifene or calcitonin nasal spray users who were compatible with regard to the potential confounders described below (see Table 1 for the variables included in the propensity score calculation).(22)

**Table 1:** Characteristics of Propensity Score–Matched Study Population in 12 Months Prior to Filling Their First Osteoporosis Drug Prescription

	Bisphosphonates	Raloxifene/calcitonin
*n*	17,028	16,787
Demographic factors		
Age, years, mean (SD)	79.9 (6.5)	80.0 (6.9)
Race, white	16,180 (95)	15,987 (95.2)
Sex, female	16,474 (96.8)	16,244 (96.8)
Health care utilization		
No. of visits, mean (SD)	10.6 (6)	10.5 (6.1)
ER visit	4,505 (26.5)	4,482 (26.7)
No. of all prescription drugs, mean (SD)	10.4 (6)	10.5 (6.1)
Hospitalization	6,089 (35.8)	6,146 (36.6)
Nursing home resident	1,882 (11.1)	1,991 (11.9)
Comorbidities		
Prior fall	2,094 (12.3)	2,119 (12.6)
Prior hip fracture	612 (3.6)	601 (3.6)
Prior vertebral fracture	1,858 (10.9)	1,890 (11.3)
BMD test	4,085 (24)	4,180 (24.9)
Hypertension	11,303 (66.4)	11,233 (66.9)
Chronic kidney disease	492 (2.9)	481 (2.9)
Chronic liver disease	207 (1.2)	191 (1.1)
Parkinson disease	586 (3.4)	598 (3.6)
Dementia	1,039 (6.1)	1,092 (6.5)
Diabetes mellitus	4,354 (25.6)	4,312 (25.7)
Congestive heart failure	3,664 (21.5)	3,728 (22.2)
Chronic obstructive pulmonary disease (COPD)	4,782 (28.1)	4,763 (28.4)
Inflammatory arthritis	1,267 (7.4)	1,258 (7.5)
Inflammatory bowel disease	241 (1.4)	226 (1.4)
Alcoholism	311 (1.8)	301 (1.8)
Comorbidity index, mean (SD)	1.9 (1.9)	2 (1.9)
Other medications		
Opioids	6,817 (40)	6,826 (40.7)
Antiepileptics	892 (5.2)	877 (5.2)
Proton pump inhibitors	4,361 (25.6)	4,441 (26.5)
Benzodiazepines	4,636 (27.2)	4,614 (27.5)
Selective serotonin reuptake inhibitors (SSRIs)	2,654 (15.6)	2,683 (16)
Warfarin	1,814 (10.7)	1,786 (10.6)
Inhaled steroid	1,389 (8.2)	1,391 (8.3)
Oral steroid	2,420 (14.2)	2,387 (14.2)

*Note:* New Jersey and Pennsylvania combined, second drug dispensing and a 90-day lag period are required. Data are presented in number (%), unless specified.

SD = standard deviation; ER = emergency room; BMD = bone mineral density.

### Drug exposures

We compared new users of oral bisphosphonate with new users of either raloxifene or calcitonin nasal spray. Oral bisphosphonates included in the study were alendronate, risedronate, and etidronate. Ibandronate was not available during the study period. Switchers between different oral bisphosphonate agents were considered as continuous users unless there was a gap between two bisphosphonate drug prescriptions of longer than 90 days.

**Fig. 1:**
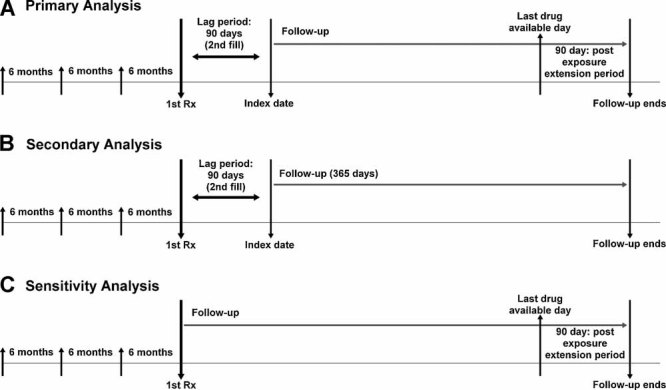
Study design. Subjects were required to have at least one claim each during the prior three 6-month intervals. For both the primary (“as treated”) and secondary (“first exposure carried forward”) analyses, follow-up began on the 91st day after filling the first prescription of either exposure of interest. The second prescription fill for the same exposure drug group was required during the 90-day lag period. For the primary analysis (*A*), we continued the follow-up until 90 days after the last drug available date. Last drug available date was calculated with a number of days of supply after the last prescription fill date. For the secondary analysis (*B*), the follow-up continued until 365 days after the index date. Patients were considered “always exposed” for the first exposure drug group during the follow-up period. In a sensitivity analysis (*C*), follow-up began at the first prescription fill and ended 90 days after the last drug available date.

For both the primary (“as treated”) and secondary (“first exposure carried forward”) analyses, in which a lag period of 90 days was required, follow-up began 91 days after filling the first prescription of either exposures of interest. The second prescription fill for the same exposure group was required during the 90-day lag period (Fig. 1).

For the primary analysis, subjects were followed up until 90 days after the last drug available date, assuming that bisphosphonates have a long duration of action. Last drug available date was calculated as the number of days of supply after the last prescription fill date. For the secondary analysis, mimicking an intention-to-treat analysis used in clinical trials, subjects were followed up for 365 days and considered “always exposed” on the basis of the first exposure regardless of drug discontinuation or switching drug during the follow-up period.

We also performed a sensitivity analysis (“as treated with no lag period”), in which the follow-up started at the date of the first prescription fill and continued until 90 days after the last drug available date (Fig. 1).

### Outcomes

We used definitions of subtrochanteric (ICD-9 820.22) and diaphyseal (ICD-9 821.0x) femur fracture based on primary hospital discharge diagnosis codes. In a recent validation study, administrative claims–based algorithms using the primary hospital discharge diagnosis codes to identify cases of subtrochanteric or diaphyseal femur fracture yielded high positive predictive values between 0.75 and 0.86.(23) The primary outcome of interest was a combined endpoint of subtrochanteric and diaphyseal femur fractures. When a patient had both subtrochanteric and diaphyseal femur fractures, it was counted as a single fracture. We also evaluated whether the outcomes were related to major trauma based on various diagnoses codes (Supplemental Table S1).

Patients were censored at the earliest time of the following events during the follow-up period: (1) occurrence of the first outcome, (2) occurrence of typical hip fracture, defined with ICD 9 820.0-820.1 and 820.8-820.9, (3) admission to a nursing home, (4) end of study period, or (5) death. Typical hip fractures were considered censoring events because most patients with hip fractures would undergo surgical repaired(24) and therefore have different risks for subsequent fractures of the femur. Owing to incomplete prescription data among nursing home residents in the study database, subjects were censored at the time of nursing home admission. Subjects who did not have any dispensing during the lag period and who had censoring events during the lag period were excluded from the analyses.

### Covariates

Patient characteristics potentially related to a future femur fracture were assessed using the data from the 12 months prior to the first prescription fill date. These characteristics included demographic factors (eg, age, sex, race, and state), calendar year, nursing home resident, health care utilization factors (eg, acute-care hospitalizations, emergency room visits, and number of physician visits and different medications), other recorded comorbidities (eg, prior falls, prior hip or vertebral fractures, BMD test, alcoholism, Parkinson disease, dementia, chronic kidney or liver disease, hypertension, diabetes mellitus, chronic obstructive pulmonary disease, heart failure, inflammatory arthritis, and inflammatory bowel disease), and use of other medications likely associated with bone metabolism or fall risks (eg, oral or inhaled glucocorticoids, anticonvulsants, benzodiazepines, selective serotonin reuptake inhibitors, beta blockers, warfarin, proton pump inhibitors, and opioids).

To quantify patients' comorbidities, we additionally calculated the Deyo-adapted Charlson Comorbidity Index based on ICD-9-CM for the 12 months prior to the first prescription fill date.(25,26) The Charlson Comorbidity Index is a summary score based on 19 major medical conditions, including myocardial infarction; pulmonary, renal, or hepatic disease; diabetes; cancer; human immunodeficiency virus infection; and so on. A score of 0 represents absence of comorbidity, and a higher score indicates a greater number of comorbid conditions. Duration of treatment with either oral bisphosphonates or raloxifene/calcitonin was assessed for subgroup analysis.

### Statistical analysis

Logistic regression models were developed to calculate the propensity score of individual patients in each state, Pennsylvania or New Jersey. The propensity score is the probability of initiating oral bisphosphonates versus either raloxifene or calcitonin nasal spray as a function of all the potential confounders listed in Table 1 and calendar year. Propensity scores were calculated at their first prescription fill date. Patients in each group (oral bisphosphonates versus raloxifene/calcitonin) then were matched 1:1 with the second decimal place of the estimated propensity scores. After the propensity score matching, subjects from the two states were pooled for all the analyses. The characteristics of patients in each group were compared before and after the propensity score matching.

The IRs of subtrochanteric and diaphyseal fractures were calculated among propensity score–matched patients in each treatment group. Cox proportional hazard analyses were used to estimate the HRs and the 95% CIs of the risk of subtrochanteric or diaphyseal femur fractures among oral bisphosphonate users compared with raloxifene/calcitonin users. Since we matched the groups on propensity scores containing potential confounders, the Cox regression models contained only a variable for the exposures of interest, with raloxifene/calcitonin as the reference exposure. We tested the proportional hazards assumption for each exposure of interest with respect to each of the fracture outcomes via the Kolmogorov supremum test.(27) We also constructed adjusted Kaplan-Meier fracture-free survival curves and inspected two-way log-rank tests. A Cox model stratified by treatment duration (<1 year, 1 to 2 years, 3 to 4 years, and 4 years or more) was used to assess the association between the risk of fracture and duration of treatment. All analyses were conducted using SAS Statistical Software Version 9.2 (SAS Institute, Inc., Cary, NC, USA).

This work was approved by the Brigham and Women's Hospital's Institutional Review Board. Data-use agreements were in place with Medicare and the state pharmacy benefit programs that supplied information for the study database. All potentially traceable personal identifiers were removed from the data before analyses to protect patient privacy.

## Results

### Cohort selection

**Fig. 2:**
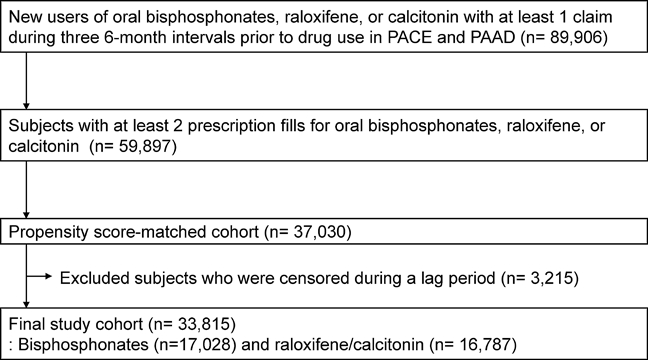
Flow diagram of cohort selection. PACE = Pennsylvania Pharmaceutical Assistance Contract for the Elderly; PAAD = New Jersey Pharmaceutical Assistance to the Aged and Disabled.

Figure 2 illustrates our cohort selection process for the primary and secondary analyses. Of 89,906 new users of oral bisphosphonates, raloxifene, or calcitonin nasal spray with at least one claim each during the prior three 6-month intervals, 59,897 subjects had at least two consecutive prescription fills. After 1:1 propensity score matching, a total of 37,030 subjects with at least two prescription fills for osteoporosis drugs were identified. We excluded 3215 patients who had a censoring event during the 90-day lag period. Our final cohort consisted of 17,028 oral bisphosphonate users and 16,787 raloxifene/calcitonin users.

For the sensitivity analysis, a total of 59,642 subjects with at least one prescription fill for osteoporosis drugs were identified after 1:1 propensity score matching.

### Patient characteristics

The baseline characteristics of propensity score–matched patients with at least two prescription fills are listed in Table 1. The mean age was 79.9 years (SD 6.5) in bisphosphonate users and 80.0 years (SD 6.9) in raloxifene/calcitonin users. Ninety-seven percent were women and 95% were white in both groups. A mean duration of follow-up was 2.13 years (SD 2.21); however, more than 4000 patients had a follow-up longer than 5 years. Approximately 84% of the subjects in the bisphosphonate group were treated with alendronate, 14% with risedronate, and 2% with etidronate. Seventy-two percent of the subjects in the raloxifene/calcitonin group were treated with calcitonin. The propensity score–matched cohorts had more similar health care utilization patterns, comorbidities, and use of other medications than the unmatched cohorts (Supplemental Table S1).

### Subtrochanteric or diaphyseal femur fractures

A total of 104 subtrochanteric or diaphyseal femur fractures was observed for our primary analysis. Only two major trauma-related fractures in the propensity score–matched cohorts were noted and excluded from the analysis. Incidence rates for subtrochanteric or diaphyseal femur fracture were calculated in the propensity score–matched cohorts (Table 2). The primary analysis estimated that there were 1.46 subtrochanteric or diaphyseal femur fractures (0.92 subtrochanteric and 0.61 diaphyseal) per 1000 person-years among the bisphosphonate users. The IRs were similar among raloxifene/calcitonin users. Similar IRs across both groups were noted in both the secondary and sensitivity analyses (Table 2).

**Table 2:** Incidence Rates (IRs) for Subtrochanteric or Diaphyseal Femur Fracture per 1000 Person-Years in the Propensity Score–Matched Population

	Bisphosphonates				Raloxifene/calcitonin			
		
Outcomes	No. of patients	No. of events	Person-years	IR^a^ (95% CI)	No. of patients	No. of events	Person-years	IR^a^ (95% CI)
Primary (as treated) analysis								
Subtrochanteric or diaphyseal femur fracture	17,028	57	39,095	1.46 (1.11–1.88)	16,787	47	32,836	1.43 (1.06–1.89)
Subtrochanteric femur fracture	17,028	36	39,098	0.92 (0.66–1.3)	16,787	34	32,836	1.04 (0.73–1.43)
Diaphyseal femur fracture	17,028	24	39,095	0.61 (0.40–0.90)	16,787	13	32,836	0.40 (0.22–0.66)
Secondary (first exposure carried forward) analysis								
Subtrochanteric or diaphyseal femur fracture	17,028	22	15,817	1.39 (0.89–2.07)	16,787	21	15,333	1.37 (0.87–2.06)
Subtrochanteric femur fracture	17,028	15	15,817	0.95 (0.55–1.53)	16,787	16	15,333	1.04 (0.62–1.66)
Diaphyseal femur fracture	17,028	8	15,817	0.51 (0.24–0.96)	16,787	5	15,333	0.33 (0.12–0.72)
Sensitivity (as treated with no lag period) analysis								
Subtrochanteric or diaphyseal femur fracture	29,780	81	58,344	1.39 (1.11–1.72)	29,743	72	46,959	1.53 (1.21–1.92)
Subtrochanteric femur fracture	29,780	41	58,347	0.70 (0.51–0.94)	29,743	45	46,936	0.96 (0.71–1.27)
Diaphyseal femur fracture	29,780	46	58,344	0.79 (0.58–1.04)	29,743	27	46,960	0.57 (0.39–0.83)

a In 1000 person-years.

**Fig. 3:**
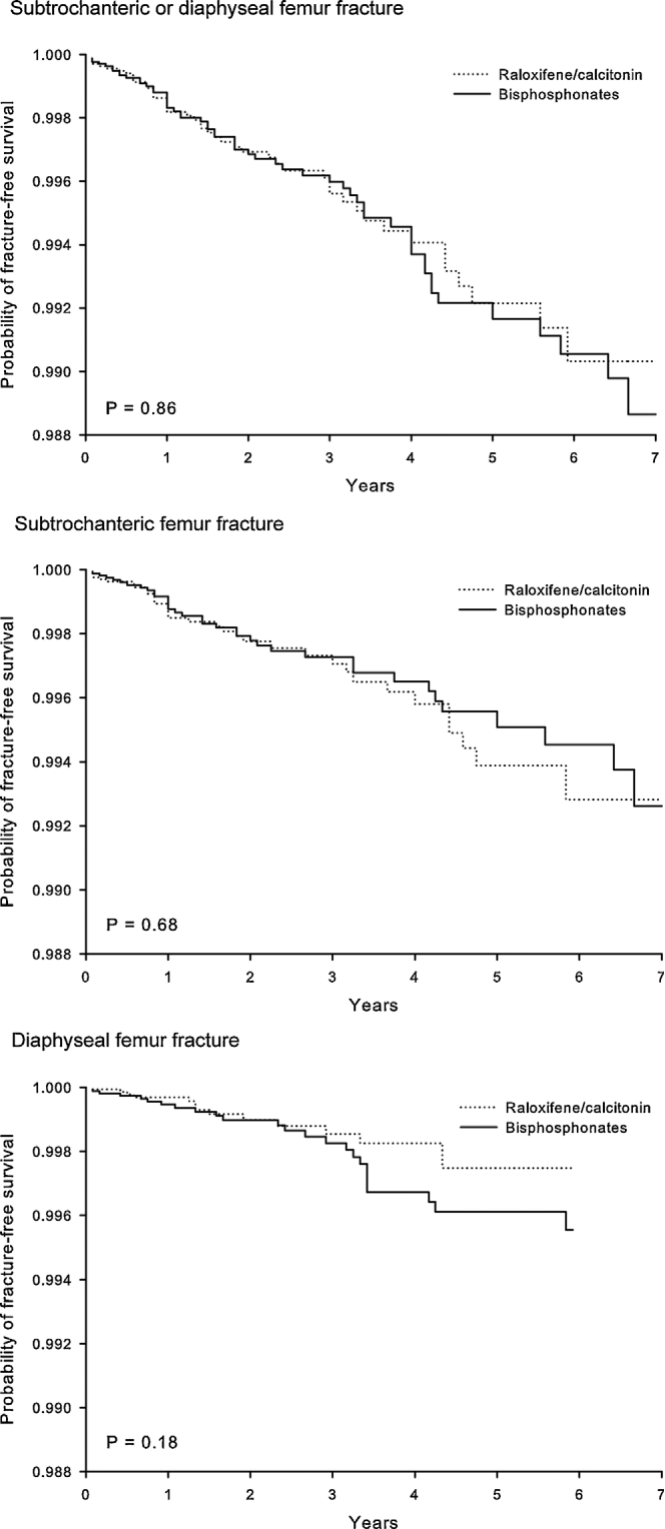
Kaplan-Meier curves for fracture-free survival in oral bisphosphonates versus raloxifene/calcitonin nasal spray.

HRs for each fracture event were estimated with Cox regression models in the propensity score–matched cohorts (Table 3). In the primary analysis, oral bisphosphonates were not associated with a significantly increased risk of subtrochanteric or diaphyseal (HR = 1.03, 95% CI 0.70–1.52), subtrochanteric (HR = 0.90, 95% CI 0.56–1.44), or diaphyseal femur fractures (HR = 1.57, 95% CI 0.80–3.09) compared with raloxifene/calcitonin. However, owing to the wide confidence interval with a relatively small number of events, we cannot exclude an increased risk for diaphyseal fractures of the femur associated with use of oral bisphosphonates. Similar results were observed in both the secondary and sensitivity analyses. For every model, the result of the Kolmogorov-type supremum test was not significant (all *p* values > .50). Therefore, the proportional-hazards assumption was not violated in our models. Figure 3 displays the Kaplan-Meier fracture-free survival curves over the follow-up period for the primary analysis. The rates of subtrochanteric or diaphyseal femur fractures did not differ meaningfully among the two groups.

**Table 3:** Hazard Ratios (95% CIs) for Subtrochanteric or Diaphyseal Femur Fracture in the Propensity Score–Matched Population

Fractures	Bisphosphonates	Raloxifene/calcitonin
Primary (as treated) analysis		
Subtrochanteric or diaphyseal femur fracture	1.03 (0.70–1.52)	1.00
Subtrochanteric femur fracture	0.90 (0.56–1.44)	1.00
Diaphyseal femur fracture	1.57 (0.80–3.09)	1.00
Secondary (first exposure carried forward) analysis		
Subtrochanteric or diaphyseal femur fracture	1.02 (0.56–1.85)	1.00
Subtrochanteric femur fracture	0.91 (0.45–1.84)	1.00
Diaphyseal fracture	1.55 (0.51–4.75)	1.00
Sensitivity (as treated with no lag period) analysis		
Subtrochanteric or diaphyseal femur fracture	0.91 (0.66–1.26)	1.00
Subtrochanteric femur fracture	0.74 (0.48–1.12)	1.00
Diaphyseal femur fracture	1.41 (0.87–2.27)	1.00

**Fig. 4:**
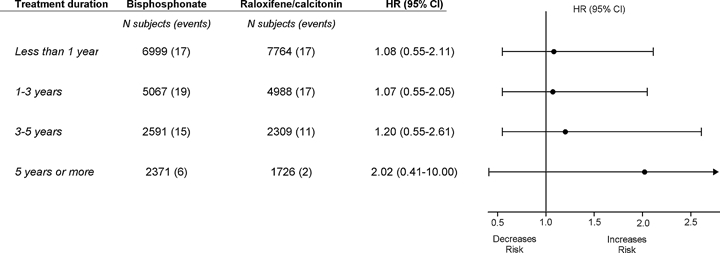
Hazard ratios (HRs) for subtrochanteric or diaphyseal femur fractures according to osteoporosis treatment duration.

Overall, no significant differences were noted between the two groups for the risk of subtrochanteric or diaphyseal fracture of the femur stratified by treatment duration (Fig. 4), although the HR was 2.02 with a wide confidence interval (95% CI 0.41–10.00) among those treated for longer than 5 years (2371 bisphosphonate users compared with 1726 raloxifene/calcitonin users).

## Discussion

Bisphosphonates are used widely for the prevention and treatment of osteopenia and osteoporosis. Common side effects such as heartburn, esophageal irritation, and musculoskeletal pain are well known, whereas few data, particularly from prospective studies with a long-term follow-up, exist on the questionable association between atypical femur fractures and the use of bisphosphonates.(13,28) In this large propensity score–matched cohort study using health care utilization data, there was no difference in the risk of subtrochanteric or diaphyseal femur fractures in bisphosphonate users compared with raloxifene/calcitonin users. Occurrence of these fractures among both bisphosphonate and raloxifene/calcitonin users was rare in this study.

The estimated IR of subtrochanteric or diaphyseal femur fractures was 1.46 per 1000 person-years among bisphosphonate users and 1.43 per 1000 person-years among raloxifene/calcitonin users. The results of the primary analysis (HR = 1.03, 95% CI 0.70–1.52) indicate that an increase in the rate of subtrochanteric or diaphyseal femur fracture associated with oral bisphosphonate uses by more than 0.74 per 1000 person-years can be excluded with a confidence level of 95%.(29)

Our study has several important implications. We compared the risk of subtrochanteric or diaphyseal femur fracture between two active osteoporosis treatment groups with the propensity score–matching method to minimize confounding by indication. In addition, we used multiple approaches in study design and analysis and obtained consistent results. Our results on the IRs of subtrochanteric or diaphyseal femur fractures are similar to those from the Danish cohort study(14) but somewhat higher than the results (0.25 per 1000 person-years) from two recent studies.(19,30) Although the IRs in our study may have been overestimated because we could not assess whether all the subtrochanteric or diaphyseal femur fractures in our study had characteristic radiographic findings of atypical fracture, such as a simple transverse fracture with cortical thickening,(6) morphologic evaluation of fractures with radiographs were not done in two other previous studies(14,30) and are available only in a subset of subjects in the secondary analyses of three RCTs.(19) Therefore, the differences in these rates probably are related to the characteristics of our study population [ie, study size, mostly female (>95%), users of osteoporosis drugs, low socioeconomic status, and a greater number of medical comorbidities and prescription drugs].

Several case series suggested a risk of atypical femur fracture particularly with long-term use of bisphosphonates.(3,6,7,9) In our subgroup analysis of 4097 patients, the relative hazard associated with long-term use of bisphosphonates (>5 years) compared with raloxifene/calcitonin use was 2.02 (95% CI 0.41–10.00). Similar results were noted in two earlier studies, although both studies were based on a much smaller number of long-term users and outcomes. There were only five subtrochanteric or diaphyseal femur fractures among 178 patients with alendronate use for longer than 6 years in the Danish cohort study (HR = 1.37, 95% CI 0.22–8.62)(14) and two atypical subtrochanteric or diaphyseal femur fractures among 662 patients with alendronate use for longer than 5 years in the Fracture Intervention Trial (FIT) Long-Term Extension trial (HR = 1.33, 95% CI 0.12–14.67).(19) Studies that did not observe statistically significant changes merit special attention to their statistical power to detect a clinically meaningful change.(31) Even though we conducted a large-scale cohort study, it is still possible that we did not have sufficient power to detect the excess risk for such a rare outcome.

In a study by Black and colleagues,(19) the number needed to treat with a bisphosphonate to observe one excess atypical femur fracture was 725 based on a hypothetical relative risk of 3.0 compared with placebo. We estimated that 450 patients would need to be treated with a bisphosphonate for more than 5 years to observe one excess subtrochanteric or diaphyseal femur fracture, assuming a hypothetical relative risk of 3.0, compared with those treated with raloxifene or calcitonin. Given the results from the FIT, which showed that treating 81 postmenopausal women with osteoporosis with alendronate for over 4 years would prevent one hip fracture,(32) the benefit clearly outweighs the risk, even with such a high hypothetical risk.

Confounding bias is the major barrier to using large administrative claims databases for pharmacoepidemiologic research. One could avoid the issue of confounding bias by conducting a RCT.(15) However, there are a number of important limitations in RCTs to study long-term safety of drugs, such as insufficient sample sizes, inadequate duration of follow-up, generalizability, ethical issues, and substantial cost. We therefore conducted a large population-based cohort study and attempted to minimize this bias by selecting new users of osteoporosis drugs and matching them based on a propensity score that included many potentially important confounders, resulting in well-balanced cohorts with respect to the measured variables in the database. However, it is possible that differences still exist between the groups, resulting in residual confounding owing to unmeasured confounders (eg, calcium and vitamin D intake, BMD, body mass index, and frailty) not included in the propensity score calculation. We included both female and male patients in this study, although the majority of patients (97%) were female. Five hundred and forty male patients in the raloxifene/calcitonin group were calcitonin users because raloxifene is indicated only in female patients.

Other important potential limitations include misclassification of exposures and outcomes. While we used pharmacy claims data, which are considered to be one of the best data sources for drug exposure, to identify the exposure in this study,(33) actual patient adherence to the medication is unknown. The outcomes in this study were identified by the diagnosis codes from administrative claims. Although the accuracy of the specific codes used in this study has been validated recently in other claims data,(23) we could not verify diagnoses of subtrochanteric or diaphyseal femur fracture based on specific radiographic characteristics in the study database. However, the impact of this misclassification bias is most likely nondifferential between bisphosphonates and raloxifene/calcitonin users.

In conclusion, we found no significant differences in the risk of either subtrochanteric or diaphyseal fractures of the femur between users of oral bisphosphonates and raloxifene/calcitonin nasal spray. Despite the large study size, however, we still had little precision in estimating the risk of subtrochanteric or diaphyseal femur fractures associated with use of bisphosphonates for more than 5 years. Thus we cannot rule out the possibility of an increased risk of these femur fractures associated with long-term use of bisphosphonates.
