# Supplementary material for: Oral Bisphosphonates and Risk of Subtrochanteric or Diaphyseal Femur Fractures in a Population-Based Cohort
Source: J Bone Miner Res. 2010 Nov 18;26(5):993–1001. doi: 10.1002/jbmr.288 (PMC3179299; doi:10.1002/jbmr.288)
Supplement: Supplementary file 1 [file jbmr0026-0993-SD1.doc]

**SUPPLEMENTAL TABLE S1.**Characteristics of Propensity Score–Unmatched Study Population in 12 Months Prior to Filling Their First Osteoporosis Drug Prescription

|  | Bisphosphonates | | Raloxifene/calcitonin |
| --- | --- | --- | --- |
| *n* | 38,148 | | 17,090 |
| *Demographic factors* | |  | |
| Age, years, mean (SD) | 79.0 (6.5) | | 80.0 (6.9) |
| Race, white | 35,570 (93.2) | | 16,284 (95.3) |
| Sex, female | 36,213 (94.9) | | 16,547 (96.8) |
| *Health care utilization* | |  | |
| No. of visit, mean (SD) | 10.4 (7) | | 10.6 (7.3) |
| ER visit | 9,015 (23.6) | | 4,634 (27.1) |
| No. of all prescription drugs, mean (SD) | 9.6 (5.6) | | 10.6 (6.1) |
| Hospitalization | 10,961 (28.7) | | 6,389 (37.4) |
| Nursing home resident | 2,972 (7.8) | | 2,120 (12.4) |
| *Comorbidities* |  | |  |
| Prior fall | 3,909 (10.3) | | 2,208 (12.9) |
| Prior hip fracture | 1,178 (3.1) | | 6,14 (3.6) |
| Prior vertebral fracture | 2,720 (7.1) | | 2,019 (11.8) |
| BMD test | 16,831 (44.1) | | 4,182 (24.5) |
| Hypertension | 26,077 (68.4) | | 11,442 (67) |
| Chronic kidney disease | 906 (2.4) | | 505 (3) |
| Chronic liver disease | 399 (1.1) | | 198 (1.2) |
| Parkinson disease | 1,118 (2.9) | | 649 (3.8) |
| Dementia | 1,701 (4.5) | | 1,175 (6.9) |
| Diabetes mellitus | 9,989 (26.2) | | 4,410 (25.8) |
| Congestive heart failure | 6,573 (17.2) | | 3,886 (22.7) |
| COPD | 9,958 (26.1) | | 4,893 (28.6) |
| Inflammatory arthritis | 2,832 (7.4) | | 1,276 (7.5) |
| Inflammatory bowel disease | 411 (1.1) | | 236 (1.4) |
| Alcoholism | 756 (2) | | 309 (1.8) |
| Comorbidity Index, mean (SD) | 1.8 (1.8) | | 2.0 (2.0) |
| *Other medications* | | | |
| Opioids | 13,286 (34.8) | | 7,045 (41.2) |
| Antiepileptics | 2,102 (5.5) | | 908 (5.3) |
| Proton pump inhibitors | 8,312 (21.8) | | 4,652 (27.2) |
| Benzodiazepines | 9,018 (23.6) | | 4,765 (27.9) |
| SSRIs | 5,185 (13.6) | | 2,806 (16.4) |
| Warfarin | 3,881 (10.2) | | 1,823 (10.7) |
| Inhaled steroid | 2,532 (6.6) | | 1,436 (8.4) |
| Oral steroid | 5,554 (14.6) | | 2,429 (14.2) |

*Note:* New Jersey and Pennsylvania combined, second drug dispensing and a 90-day lag period are required. Data are presented as number (%) unless otherwise specified. SD = standard deviation; ER = emergency room; BMD = bone mineral density; COPD = chronic obstructive pulmonary disease; SSRI = selective serotonin reuptake inhibitors.
